# A New Hand Piece

**Published:** 1874-11

**Authors:** 


### A NEW HAND PIECE.

We are using upon the Morrison engine a new hand piece that in one respect is superior to any one we have used before: viz, the ease with which the burrs and drills are fastened in it, and the firmness with which they are held, this is done by the retaining point for the drill being slit longitudinally from the end, the drill placed in the holder, is made firm in place by a sliding band or ring being drawn tightly on to the point.

This instrument was designed and constructed by Drs. Evans & Edson, of Toledo, O.

